# High Eradication Rate of a Potassium‐Competitive Acid Blocker‐Based Minocycline, Furazolidone, and Bismuth Regimen for Refractory *Helicobacter pylori* Infection: A Real‐World Study

**DOI:** 10.1111/hel.70128

**Published:** 2026-05-16

**Authors:** Ji‐Yan Li, Ying‐Ying Han, Dong‐Yan Li, Jing‐Mei Liu, Zhen‐Zhen Zhou, Ya‐Ni Zhou, Lin Tuo, Xiao‐Jiao Yao, Mei Liu, Ge Wang, Pei‐Yuan Li

**Affiliations:** ^1^ Division of Gastroenterology Tongji Hospital, Tongji Medical College, Huazhong University of Science and Technology Wuhan China; ^2^ Division of Pharmacy Tongji Hospital, Tongji Medical College, Huazhong University of Science and Technology Wuhan China; ^3^ Health Management Center Tongji Hospital, Tongji Medical College, Huazhong University of Science and Technology Wuhan China

**Keywords:** bismuth, furazolidone, minocycline, P‐CAB, refractory *Helicobacter pylori* infection

## Abstract

**Background:**

Management of refractory *Helicobacter pylori* ( *H. pylori* ) infection remains challenging due to increasing antibiotic resistance and limited effective rescue options. Minocycline and furazolidone retain low resistance rates, while potassium‐competitive acid blockers (P‐CABs) provide potent and sustained acid suppression. This study aimed to evaluate the efficacy and safety of a 14‐day P‐CAB‐based regimen combining minocycline, furazolidone, and bismuth in patients with refractory *H. pylori* infection.

**Methods:**

From January 2023 to October 2025, clinical data were collected from patients with refractory *H. pylori* infection who received the above regimen at Tongji Hospital, Wuhan, China. Eradication was assessed by a urea breath test performed at least 4 weeks after treatment. Adverse events during treatment were recorded.

**Results:**

A total of 368 patients were included in modified intention‐to‐treat analysis. Of them, 183 (49.7%) had two previous eradication failures, 165 (44.8%) had three to four failures, and 20 (5.4%) had five or more failures. Among the 143 patients who underwent antibiotic resistance gene testing, the resistance rates to clarithromycin and quinolones were 96.5% and 79.7%, respectively. The *H. pylori* eradication rate was 92.7% (95% CI 89.5%–94.9%) in modified intention‐to‐treat analysis and 96.4% (95% CI 93.8%–97.9%) in per‐protocol analysis. Adverse events occurred in 25.8% of patients and were predominantly mild to moderate, with dizziness (9.5%), nausea (5.2%), and abdominal distension (5.2%) most common. Poor compliance (OR = 18.14, 95% CI 6.39–51.51) and peptic ulcer (OR = 4.34, 95% CI 1.27–14.82) were independently associated with eradication failure.

**Conclusions:**

The 14‐day regimen of P‐CAB, minocycline, furazolidone, and bismuth is highly effective and well tolerated in patients with refractory *H. pylori* infection. This regimen represents a promising empirical rescue therapy, particularly in settings where antimicrobial susceptibility testing is unavailable.

## 1 Introduction

*Helicobacter pylori* ( *H. pylori* ) infection is a major etiological factor in a wide spectrum of gastric diseases, ranging from chronic gastritis and peptic ulcer disease to gastric adenocarcinoma and mucosa‐associated lymphoid tissue lymphoma. Additionally, accumulating evidence has linked *H. pylori* infection to multiple extra‐gastric disorders, underscoring its substantial impact on global health [1, 2]. Although improvements in sanitation and public health measures have led to a gradual decline in *H. pylori* prevalence in some regions [3], the incidence rate in developing countries still remains as high as 40.9%–79.5% [4] and continues to pose a significant clinical and economic burden. The effectiveness of *H. pylori* eradication therapy has been increasingly compromised by the rapid emergence of antibiotic resistance. Globally, resistance rates to clarithromycin, levofloxacin, and metronidazole have exceeded the critical threshold of 15% in most regions, rendering many standard regimens suboptimal [5]. Although amoxicillin has traditionally been considered a low‐resistance antibiotic and remains a cornerstone of first‐ and second‐line therapies, emerging evidence suggests that secondary resistance to amoxicillin increases significantly to over 20% after repeated eradication failures in some areas [6, 7]. Consequently, refractory *H. pylori* infection, generally defined as persistent infection after at least two standard eradication attempts [8], has become an increasingly common and challenging clinical problem, with limited effective therapeutic options.

For refractory *H. pylori* infection, antibiotics with persistently low resistance rates are of particular interest. Tetracycline and furazolidone have demonstrated stable antimicrobial activity against *H. pylori* , with resistance rates remaining low even after multiple eradication failures [9]. However, the clinical use of tetracycline is limited in many countries due to its adverse event profile and poor availability. Minocycline, a semi‐synthetic tetracycline derivative, has emerged as a potential alternative. It offers similar antimicrobial mechanisms with improved pharmacokinetic properties, including a longer half‐life, higher bioavailability, and enhanced tissue penetration [10]. Several recent studies have suggested that minocycline‐containing regimens may achieve eradication rates comparable to those of tetracycline‐based therapies, with acceptable safety profiles [11, 12, 13, 14].

Furazolidone is another antibiotic that has retained high sensitivity against *H. pylori* and is used in some parts of Asia and Latin America. Despite concerns regarding its potential carcinogenicity based on animal studies, current clinical evidence has not demonstrated definitive teratogenic or clastogenic effects in humans [15]. In regions with high antimicrobial resistance and limited access to alternative agents, furazolidone continues to play an important role in rescue therapy. Nevertheless, data on its safety and efficacy in combination with newer acid‐suppressive agents remain limited.

Beyond antibiotic selection, adequate acid suppression is a critical determinant of *H. pylori* eradication success. Profound and sustained elevation of intragastric pH enhances antibiotic stability and bacterial susceptibility [16]. Potassium‐competitive acid blockers (P‐CABs), such as vonoprazan and tegoprazan, provide more rapid, potent, and consistent acid suppression than conventional proton pump inhibitors (PPIs) [17, 18]. P‐CABs‐based regimens have demonstrated superior or non‐inferior eradication rates compared with PPIs‐based therapies [19] and have been increasingly endorsed by international guidelines [8, 20, 21, 22]. However, evidence supporting the use of P‐CABs in refractory *H. pylori* infection, particularly in combination with minocycline and furazolidone, remains scarce.

At present, clinical data on the use of the novel rescue regimens that integrate P‐CABs with low‐resistance antibiotics minocycline and furazolidone for the treatment of refractory *H. pylori* infection are rare. In addition, real‐world evidence on the safety of furazolidone in clinical practice remains limited. Herein, the present real‐world study aimed to evaluate the efficacy and safety of a 14‐day regimen combining P‐CABs, minocycline, furazolidone, and bismuth (PMFB) in a relatively large population of patients with refractory *H. pylori* infection. This study seeks to provide practical evidence to inform rescue treatment strategies, particularly in regions where antimicrobial susceptibility testing is unavailable or impractical.

## 2 Materials and Methods

### 2.1 Study Design

This was a retrospective, observational, real‐world study conducted at Tongji Hospital, Tongji Medical College, Huazhong University of Science and Technology in Wuhan, central China. The study protocol was approved by the Institutional Ethics Committee of Tongji Hospital (TJ‐IRB202512166).

### 2.2 Patients

Patients diagnosed with refractory *H. pylori* infection who attended Tongji Hospital between January 2023 and October 2025 and received PMFB therapy were screened for inclusion. Patients were included if they met all of the following criteria: (1) documented history of ≥ 2 continuous failed standard *H. pylori* eradication attempts; (2) receipt of a 14‐day PMFB regimen. Patients were excluded if follow‐up UBT was not performed or yielded indeterminate results.

The diagnosis of *H. pylori* infection and assessment of eradication outcome were performed using either the ^13^C‐ or ^14^C‐UBT, in accordance with standard clinical practice. Patients were instructed to discontinue antibiotics, bismuth compounds, and acid‐suppressive agents prior to testing, as recommended by current guidelines [23].

### 2.3 Antimicrobial Resistance Gene Testing

A subset of patients underwent gastroscopy before treatment, during which gastric mucosal biopsy specimens were obtained. Use the Clarithromycin, Quinolone‐Resistant and Non‐Resistant *Helicobacter pylori* Nucleic Acid Amplification Test Kit (Jiangsu Mole Bioscience Co. Ltd., Taizhou, China), Real‐time polymerase chain reaction (PCR) was performed to detect: (1) *H. pylori* 16S rRNA; (2) 23S rRNA gene mutations related to clarithromycin resistance; (3) gyrA gene mutations related to quinolone antibiotic resistance.

### 2.4 Treatment

The PMFB therapy included P‐CAB (vonoprazan 20 mg or tegoprazan 50 mg) twice daily, minocycline 100 mg twice daily, furazolidone 100 mg twice daily, and colloidal bismuth pectin 200 mg twice daily or 150 mg three times daily. The course of treatment was 14 days.

### 2.5 Outcomes

The primary outcome was *H. pylori* eradication rate. Given the real‐world nature of the study and incomplete post‐treatment testing in some patients, a conventional intention‐to‐treat (ITT) analysis was not feasible. Therefore, eradication efficacy was assessed using modified intention‐to‐treat (mITT) and per‐protocol (PP) analyses.

Secondary outcomes included adverse events, compliance, and factors influencing eradication rates. Adverse events were graded as mild (transient discomfort not affecting daily activities), moderate (symptoms partially interfering with daily activities), or severe (symptoms severely affecting daily activities or requiring treatment discontinuation). Types of adverse events were recorded individually, with multiple events in a single patient counted as distinct occurrences. Compliance was calculated as (actual pills taken/protocol‐defined pills prescribed) × 100%, with ≥ 80% defined as good adherence.

### 2.6 Data Collection

In the initial screening phase, a researcher identified all *H. pylori* ‐infected patients treated with PMFB therapy from the Hospital Information System of Tongji Hospital. Subsequently, two independent reviewers systematically extracted structured data from these patients' medical histories, capturing demographic characteristics, underlying diseases, previous treatment status of *H. pylori* infection, relevant examination, and follow‐up documentation. Patients with ≥ 2 prior eradication attempts were then isolated by a dedicated researcher for telephone follow‐up. This protocol included: 1. Verification of treatment compliance and adverse events; 2. Collection of post‐therapy UBT results (timing/methodology/outcomes); 3. Exclusion of cases with incomplete UBT documentation. Finally, another two investigators conducted verifications to ensure data completeness and accuracy across all critical variables.

### 2.7 Statistical Analysis

SPSS 26.0 (IBM Corp. Armonk, NY, USA) was used for data analysis. Continuous variables were described as median (interquartile range), and categorical data were described as absolute numbers and percentage frequencies. Comparisons between groups were made using the chi‐square test for categorical variables and the Mann–Whitney test for continuous variables. Univariate analysis was performed to explore significant predictive variables for *H. pylori* eradication. To avoid missing potential factors, all variables with a *p* value < 0.10 were included in multivariate logistic regression for further analysis of independent influencing factors. Statistical significance was defined as a two‐sided *p* value < 0.05.

## 3 Results

### 3.1 Characteristics

A total of 405 patients met the inclusion criteria, among which 37 patients were excluded due to the lack of follow‐up test results. 368 patients were included in the mITT analysis. The demographic information and clinical characteristics of patients with successful and failed eradication are shown Table 1.

**TABLE 1:** The demographic information and clinical characteristics of patients.

Factors	Successful eradication (*n* = 341)	Failed eradication (*n* = 27)	*p*
Sex (male:female)	129:212	10:17	0.935
Age, y	51 (43–58)	51 (46–55)	0.837
BMI, kg/m^2^	23.2 (20.2–26.0)	22.8 (19.8–23.9)	0.210
BSA, m^2^	1.65 (1.55–1.75)	1.60 (1.57–1.70)	0.325
Smoking	63 (18.5%)	6 (22.2%)	0.631
Drinking (≥ 1 time per month)	60 (17.6%)	5 (18.5%)	0.904
Combined chronic diseases	120 (35.2%)	8 (29.6%)	0.559
Liver diseases	11 (3.2%)	3 (11.1%)	0.124
Kidney diseases	1 (0.3%)	0	1.000
Family history of gastric cancer	12 (3.5%)	1 (3.7%)	1.000
History of penicillin allergy	25 (7.3%)	0	0.239
Digestive symptoms	187 (54.8%)	13 (48.1%)	0.502
Abdominal distension	95 (27.9%)	7 (25.9%)	0.829
Acid reflux	67 (19.6%)	7 (25.9%)	0.433
Belching	65 (19.1%)	2 (7.4%)	0.211
Abdominal pain	58 (17.0%)	5 (18.5%)	0.841
Upper abdominal discomfort	53 (15.5%)	2 (7.4%)	0.389
Heartburn	41 (12.0%)	3 (11.1%)	1.000
Nausea	9 (2.6%)	3 (11.1%)	0.068
Endoscopy diagnosis	271 (79.5%)	21 (77.8%)	0.834
Non‐atrophic gastritis	173/271 (63.8%)	10/21 (47.6%)	0.139
Atrophic gastritis	157/271 (57.9%)	8/21 (38.1%)	0.077
Peptic ulcer	27/271 (10.0%)	6/21 (28.6%)	0.009
Gastric polyp	18/271 (6.6%)	3/21 (14.3%)	0.386
Reflux esophagitis	12/271 (4.4%)	2/21 (9.5%)	0.601
Antimicrobial resistance gene testing	133 (39.0%)	10 (37.0%)	0.840
Clarithromycin	128/133 (96.2%)	10/10 (100%)	1.000
Quinolones	104/133 (78.2%)	10/10 (100%)	0.213
Number of previous failed eradication treatments			0.791
2	168 (49.3%)	15 (55.6%)	
3–4	154 (45.2%)	11 (40.7%)	
≥ 5	19 (5.6%)	1 (3.7%)	
Previously used bismuth quadruple therapy	325 (95.3%)	27 (100%)	0.619
Previously used triple therapy	68 (19.9%)	3 (11.1%)	0.387
Previously used high‐dose dual therapy	36 (10.6%)	0	0.092
Previously used P‐CABs‐containing therapy	48 (14.1%)	0	0.035
Previously used tetracycline or minocycline‐containing therapy	11 (3.2%)	1 (3.7%)	1.000
Previously used furazolidone‐containing therapy	31 (9.1%)	0	0.149
P‐CAB used this time			0.228
Vonoprazan	304 (89.1%)	22 (81.5%)	
Tegoprazan	37 (10.9%)	5 (18.5%)	
The dosage of colloidal bismuth pectin used this time			0.829
200 mg twice daily	278 (81.5%)	23 (85.2%)	
150 mg three times daily	63 (18.5%)	4 (14.8%)	
With adverse events	81 (23.8%)	14 (51.9%)	0.001
Good compliance	323 (94.7%)	12 (44.4%)	< 0.001

Abbreviations: BMI, body mass index; BSA, body surface area; P‐CAB, potassium‐competitive acid blocker.

Among the included patients, 183 (49.7%) had experienced two previous eradication failures, 165 (44.8%) had three to four failures, and 20 (5.4%) had five or more failures. Most patients had previously received bismuth‐containing quadruple therapy (95.3%), and a substantial proportion had been exposed to amoxicillin‐containing regimens (89.4%). In this treatment, vonoprazan was administered to 326 patients (88.6%), while 42 patients (11.4%) received tegoprazan.

292 patients underwent gastroscopy before treatment, and 143 patients underwent antimicrobial resistance gene testing. The resistance rates of clarithromycin and quinolones were 96.2% and 78.2% respectively. To assess potential bias, differential analyses were performed on the baseline information that may affect whether patients underwent gastroscopy or antimicrobial resistance gene testing (Tables S2 and S3). Patients with digestive symptoms were more likely to have undergone these examinations. In addition, gastroscopy was more likely to be performed in older patients, smokers, and those with a higher number of previous eradication treatments (≥ 3 times). It should be noted that there was no statistical significance in the differences between whether patients underwent antimicrobial resistance gene testing and the number of previous treatments, previous medication regimens, or other factors besides symptoms.

### 3.2 *H. pylori* Eradication Rate

In the mITT analysis, the *H. pylori* eradication rate was 92.7% (341/368; 95% CI, 89.5%–94.9%). After excluding 33 patients with poor compliance, 335 patients were included in the PP analysis, yielding an eradication rate of 96.4% (323/335; 95% CI, 93.8%–97.9%).

Eradication efficacy remained consistently high across subgroups stratified by the number of previous eradication failures, the type of P‐CAB used, the dosage of colloidal bismuth pectin used, and whether antimicrobial resistance gene testing was performed, with no statistically significant differences observed (Table S1).

### 3.3 Adverse Events and Compliance

Overall, 95 patients (25.8%) experienced at least one adverse event during treatment (Table 2). Most adverse events were mild (20.7%) or moderate (4.3%) in severity. Only three patients (0.8%) experienced severe adverse events, including severe nausea with dizziness, vomiting, and rash, leading to treatment discontinuation.

**TABLE 2:** Adverse events and compliance.

Safety and compliance	Cases (proportion)
Patients with adverse events	95 (25.8%)
Mild	76 (20.7%)
Moderate	16 (4.3%)
Severe	3 (0.8%)
Variety of adverse events	
Dizziness	35 (9.5%)
Nausea	19 (5.2%)
Abdominal distension	19 (5.2%)
Abdominal pain	11 (3.0%)
Vomiting	10 (2.7%)
Diarrhea	10 (2.7%)
Fatigue	8 (2.2%)
Rash	5 (1.4%)
Headache	5 (1.4%)
Insomnia	5 (1.4%)
Discontinued drugs because of adverse events	6 (1.6%)
Good compliance	335 (91.0%)

The most frequently reported adverse events were dizziness (9.5%), nausea (5.2%), abdominal distension (5.2%), abdominal pain (3.0%), vomiting (2.7%), diarrhea (2.7%), and fatigue (2.2%). All adverse events resolved after treatment discontinuation or appropriate symptomatic management. There were no significant statistical differences in the incidence of adverse events among patients using different P‐CABs (Table S4) and different doses of colloidal bismuth pectin (Table S5).

A total of six patients (1.6%) discontinued therapy due to adverse events. Overall, 335 patients (91.0%) demonstrated good compliance.

### 3.4 Risk Factors Influencing Efficacy

Variables with *p* < 0.100 in univariate analysis (Table 1), including nausea symptoms, atrophic gastritis, peptic ulcer disease, prior high‐dose dual therapy, prior P‐CABs‐containing therapy, adverse events, and compliance were entered into the multivariate logistic regression model (Table 3). Analysis revealed no evidence of serious multicollinearity among the independent variables in the logistic regression model, as assessed by tolerance, variance inflation factor (VIF), condition index, and variance proportions (Tables S6 and S7). Figure S1 displays the receiver operating characteristic (ROC) curve for the model, achieving an area under the curve (AUC) of 0.869 (95% CI, 0.790–0.948).

**TABLE 3:** Multivariate analysis of factors influencing the eradication rate.

Variables	*p*	OR	95% CI
Symptoms of nausea	0.171	3.31	0.60–18.40
Atrophic gastritis	0.153	0.48	0.17–1.32
Peptic ulcer	0.019	4.34	1.27–14.82
Previously used high‐dose dual therapy	0.999	0	0
Previously used P‐CABs‐containing therapy	0.988	0	0
Adverse events	0.387	1.57	0.57–4.36
Poor compliance	< 0.001	18.14	6.39–51.51

Abbreviation: P‐CAB, potassium‐competitive acid blocker.

In multivariate analysis, poor compliance emerged as the strongest independent predictor of eradication failure (OR = 18.14; 95% CI, 6.39–51.51; *p* < 0.001). Peptic ulcer disease was also independently associated with reduced eradication efficacy (OR = 4.34; 95% CI, 1.27–14.82; *p* = 0.019). Adverse events were not independently associated with eradication failure after adjustment for compliance and other covariates.

## 4 Discussion

In this real‐world study, we demonstrated that a novel 14‐day bismuth‐containing quadruple regimen integrating P‐CABs with two low‐resistance antibiotics (minocycline and furazolidone) achieved a high eradication rate and acceptable safety profile in patients with refractory *H. pylori* infection. Notably, despite multiple prior eradication failures and a high prevalence of resistance to clarithromycin and quinolones, this regimen yielded eradication rates exceeding 90% in the mITT analysis and 95% in the PP analysis. Importantly, its efficacy was largely independent of the number or type of previous eradication failures, a key advantage that distinguishes it from existing regimens and supports its robustness as a rescue strategy. Adverse events were predominantly mild to moderate, and treatment discontinuation due to intolerance was infrequent, indicating good overall tolerability. Thus, these findings suggest that this innovative P‐CAB‐based minocycline‐furazolidone‐bismuth quadruple regimen represents an effective and practical empirical option for refractory *H. pylori* infection, addressing a critical unmet need in routine clinical practice.

It is estimated that 10%–20% of patients develop refractory *H. pylori* infection [24], posing a significant clinical challenge, particularly in regions with a high incidence of gastric cancer, such as East Asia [25]. Currently recommended rescue strategies include bismuth‐containing quadruple regimens based on tetracycline, metronidazole, amoxicillin or furazolidone, rifabutin‐based triple therapy, and regimens guided by antimicrobial susceptibility testing [8, 20, 21, 26, 27]. However, several unresolved issues continue to compromise the effectiveness and feasibility of these approaches.

A major challenge in refractory *H. pylori* infection is the progressive accumulation of antibiotic resistance with repeated treatment exposure. Although the primary resistance rate of amoxicillin remains low worldwide, generally below 5% outside Africa [28], its role in rescue therapy has become increasingly controversial. In the present study, 89.4% of patients had previously received amoxicillin‐containing regimens. Emerging evidence indicates that secondary resistance to amoxicillin may rise substantially after multiple eradication failures, reaching 24.5%–34.1% in third‐line and subsequent treatments [6, 7]. Repeated exposure to amoxicillin, particularly when combined with antibiotics to which *H. pylori* is already resistant, may promote the selection of resistant strains. In addition, methodological heterogeneity in susceptibility testing, including the use of disk diffusion methods and non‐standardized breakpoints, may have led to underestimation of amoxicillin resistance in earlier studies [29, 30]. Taken together, these findings suggest that amoxicillin resistance should not be overlooked when selecting rescue regimens for refractory infection.

Safety and tolerability represent another major limitation of existing rescue therapies. Tetracycline, a classical component of bismuth quadruple therapy, is associated with frequent gastrointestinal adverse events and the risk of superinfection, resulting in strict regulatory control and limited availability in China and other regions [31]. Metronidazole requires high daily doses (1.5–2 g per day) to overcome resistance, which further increases the burden of adverse effects [8, 26]. Rifabutin‐based regimens are constrained by concerns regarding bone marrow suppression and their potential impact on tuberculosis control [32]. Although the safety of furazolidone has been questioned due to carcinogenicity observed in animal studies, it continues to be widely used in certain regions and has achieved favorable eradication rates. Importantly, accumulating clinical evidence suggests that the therapeutic value of furazolidone in *H. pylori* eradication may have been underestimated, particularly in the context of multidrug resistance [33].

The limited accessibility of antimicrobial susceptibility testing further complicates the management of refractory *H. pylori* infection. Culture‐based testing is labor‐intensive, costly, and technically demanding, while PCR‐based resistance gene detection requires specialized equipment and is often unavailable in primary or resource‐limited settings. Moreover, reliable genotype–phenotype concordance has been established mainly for clarithromycin and quinolones. In patients with refractory infection, resistance rates to these agents are exceedingly high. In the present study, clarithromycin and quinolone resistance reached 96.5% and 79.7%, respectively. Moreover, there was no statistically significant difference in the history of clarithromycin and levofloxacin use between the untested group and the tested group. It can be inferred that in patients without antimicrobial resistance gene testing, the resistance rates to these two antibiotics likely reached comparable levels. Accordingly, major guidelines, including the AGA Expert Review, the Taipei Consensus, and the ACG Clinical Guideline, discourage reuse of macrolides and quinolones in rescue therapy unless susceptibility is confirmed [21, 26, 27]. Under these circumstances, the clinical utility of resistance gene testing in refractory infection may be limited, reinforcing the need for effective empirical treatment strategies.

Against this background, the PMFB regimen represents a rational and practical therapeutic option. Previous studies have reported eradication rates of 92.7% (mITT analyses) and 94.5% (PP analyses) for tetracycline and furazolidone‐containing bismuth quadruple therapy in third‐line treatment^33^. The results are highly consistent with those observed in the present study. The favorable efficacy of PMFB can be largely attributed to the use of minocycline and furazolidone, both of which retain high activity against *H. pylori* , with resistance rates generally below 3% [13, 28, 34] and no clear increase with repeated eradication attempts [7]. Both antibiotics have demonstrated favorable efficacy in *H. pylori* eradication. Evidence demonstrates that the eradication rate of the quadruple therapy containing minocycline is slightly higher but without statistical difference compared with the quadruple therapy without minocycline (83.8% vs. 82.4% for ITT analyses; 91.1% vs. 89.3% for PP analyses) [10]. Other studies have shown that the furazolidone‐containing therapies is superior to that of other antibiotics‐containing therapies (81.1% vs. 75.4% for ITT analyses; 86.6% vs. 80.1% for PP analyses) [35]. Meanwhile, the incorporation of P‐CABs provides rapid, potent, and sustained acid suppression, thereby enhancing antibiotic stability and antibacterial activity. Compared with PPIs‐based regimens, P‐CABs‐based therapies have demonstrated superior or comparable eradication rates, particularly in difficult‐to‐treat populations [16, 19, 36]. In addition, the bacteriostatic effect of bismuth increases the sensitivity of *H. pylori* to antibiotics [37]. Compared with the triple therapy, the bismuth quadruple therapy can achieve a higher eradication rate and is recommended for the treatment of refractory *H. pylori* infection [38, 39, 40].

From a safety perspective, the PMFB regimen demonstrated an acceptable tolerability profile. The overall incidence of adverse events was comparable to that reported in previous studies of bismuth‐containing quadruple therapies, and most events were mild to moderate in severity. Dizziness was relatively more frequent, which may be explained by the strong tissue penetration of minocycline and its effects on the central nervous system [41]. Importantly, while adverse events and eradication failure were correlated in univariate analysis, this association was no longer significant after adjustment for compliance, indicating that adverse events may impair treatment outcomes indirectly by reducing adherence. These findings highlight the importance of patient education and adherence support. Adjunctive strategies, such as vitamin B6 supplementation, which have been shown to alleviate neurological and gastrointestinal adverse reactions, may further improve tolerability and compliance in clinical practice [42].

The study found that peptic ulcer disease emerged as a potential factor associated with reduced eradication efficacy. This contradicts previous studies indicating that *cagA*‐positive strains are risk factors for ulcers and protective factors for eradication therapy [43]. However, some studies have also provided some possible mechanisms for the reduced eradication rate in ulcer patients: 1. Bacterial load: The average density of *H. pylori* in the gastric antrum of patients with duodenal ulcer is several times that of other patients [44]. Meanwhile, high density of *H. pylori* might be unfavorable for complete eradication [45, 46]. 2. Virulence factors: *H. pylori* with *vacA* m1 genotype is associated with severe gastritis and ulcers, and significantly reduces the eradication rate compared with m2 genotype [43]. 3. Metaplasia: Gastric metaplasia significantly increases the risk of duodenal ulcer [47] and reduces the eradication rate of *H. pylori* , presumably related to the micro‐environment formed by intestinal metaplasia [43, 48]. 4. Inflammation and angiogenesis: *H. pylori* stimulate cytokines such as interleukin‐8 (IL‐8), IL‐6, and tumor necrosis factor‐α, which inhibits angiogenic process in ulcers [49]. Therefore, the local drug concentration might decrease, affecting the treatment. Additionally, the limited sample size of ulcer patients in this study necessitates further investigation in larger prospective studies.

Notably, the present study demonstrates the broad applicability of the PMFB regimen in refractory *H. pylori* infection. Eradication efficacy was not significantly influenced by the number of previous eradication failures, prior treatment regimens, previous exposure to similar drugs, or the performance of gastroscopy or resistance gene testing. These results support the use of the PMFB regimen as an empirical rescue therapy, particularly for patients without peptic ulcer disease and in settings where culture‐based or molecular susceptibility testing is not readily available.

Several limitations of this study should be acknowledged. First, its retrospective, single‐center design may introduce selection bias and limit generalizability. However, the real‐world nature of the study allows for the inclusion of a large and clinically representative patient population, enhancing the practical relevance of the findings. The adverse events and compliance of patients might be affected by recall bias. The results of gastroscopy and antimicrobial resistance gene testing might be affected by selection bias. However, we conducted a difference analysis on the available demographic and clinical information, and this selection bias also reflected the tendency of choosing whether to conduct examinations in the real world. Second, systematic laboratory monitoring and long‐term follow‐up data were limited. Although patients with chronic liver or kidney disease did not experience disease exacerbation during treatment, future prospective studies incorporating comprehensive safety assessments and extended follow‐up are needed. Finally, multicenter studies across different geographic regions would be valuable to further validate the efficacy and safety of the PMFB regimen in diverse populations.

## 5 Conclusion

This real‐world study confirms that the PMFB regimen demonstrates high efficacy and acceptable safety in the treatment of refractory *H. pylori* infection. Poor compliance and peptic ulcer disease may adversely affect eradication outcomes. Given its robust performance across multiple clinical subgroups and its feasibility in the absence of antimicrobial susceptibility testing, the PMFB regimen may thus represent a promising and valuable empirical rescue option for patients with multiple prior eradication failures.

## Author Contributions

Conceptualization, J.‐Y.L., Y.‐Y.H., G.W. and P.‐Y.L.; methodology, J.‐Y.L. and Y.‐Y.H.; writing – original draft preparation, J.‐Y.L.; project administration, validation, supervision, writing – review and editing, G.W. and P.‐Y.L.; formal analysis, Y.‐N.Z., L.T. and X.‐J.Y.; investigation, J.‐M.L., Z.‐Z.Z., M.L.; data curation, D.‐Y.L.; funding acquisition, P.‐Y.L. All authors read and agreed to the final manuscript.

## Funding

This research was funded by the Tongji Hospital Clinical Research Fund (2024TJCR01).

## Conflicts of Interest

The authors declare no conflicts of interest.

## Supporting information


**Figure S1:** The Receiver Operating Characteristic (ROC) curve of the logistic regression model.
**Table S1:** Eradication rates in patients of different subgroups.
**Table S2:** The demographic information and clinical characteristics of patients who underwent and did not undergo gastroscopy.
**Table S3:** The demographic information and clinical characteristics of patients with and without tested antimicrobial resistance genes.
**Table S4:** Adverse events of vonoprazan‐based versus tegoprazan‐based regimen.
**Table S5:** Adverse events of different doses of colloidal bismuth pectin.
**Table S6:** Tolerance and variance Inflation Factor (VIF) in the regression model.
**Table S7:** Collinearity diagnostics.

## Data Availability

The data that support the findings of this study are available on request from the corresponding author. The data are not publicly available due to privacy or ethical restrictions.
