# Supplementary material for: High Eradication Rate of a Potassium‐Competitive Acid Blocker‐Based Minocycline, Furazolidone, and Bismuth Regimen for Refractory Helicobacter pylori Infection: A Real‐World Study
Source: Helicobacter. 2026 May 16;31:e70128. doi: 10.1111/hel.70128 (PMC13179562; doi:10.1111/hel.70128)
Supplement: Supplementary file 1 — Figure S1: The Receiver Operating Characteristic (ROC) curve of the logistic regression model. Table S1: Eradication rates in patients of different subgroups. Table S2: The demographic information and clinical characteristics of patients who underwent and did not undergo gastroscopy. Table S3: The demographic information and clinical characteristics of patients with and without tested antimicrobial resistance genes. Table S4: Adverse events of vonoprazan‐based versus tegoprazan‐based regimen. Table S5: Adverse events of different doses of colloidal bismuth pectin. Table S6: Tolerance and variance Inflation Factor (VIF) in the regression model. Table S7: Collinearity diagnostics. [file HEL-31-e70128-s001.docx]

**Table S1. Eradication rates in patients of different subgroups**

| **Subgroup** | **Eradication rate** | ***P*** |
| --- | --- | --- |
| **mITT analysis** |  |  |
| Number of previous failed eradication treatments |  | 0.791 |
| 2 | 91.8% (168/183) |  |
| 3-4 | 93.3% (154/165) |  |
| ≥5 | 95.0% (19/20) |  |
| P-CAB used this time |  | 0.228 |
| Vonoprazan | 93.3% (304/326) |  |
| Tegoprazan | 88.1% (37/42) |  |
| The dosage of colloidal bismuth pectin used this time |  | 0.829 |
| 200 mg twice daily | 92.4% (278/301) |  |
| 150 mg three times daily | 94.0% (63/67) |  |
| Whether to perform antimicrobial resistance gene testing |  | 0.840 |
| Yes | 93.0% (133/143) |  |
| No | 92.4% (208/225) |  |
| **PP analysis** |  |  |
| Number of previous failed eradication treatments |  | 0.697 |
| 2 | 96.3% (158/164) |  |
| 3-4 | 96.1% (147/153) |  |
| ≥5 | 100.0% (18/18) |  |
| P-CAB used this time |  | 1.000 |
| Vonoprazan | 96.3% (288/299) |  |
| Tegoprazan | 97.2% (35/36) |  |
| The dosage of colloidal bismuth pectin used this time |  | 1.000 |
| 200 mg twice daily | 96.3% (263/273) |  |
| 150 mg three times daily | 96.8% (60/62) |  |
| Whether to perform antimicrobial resistance gene testing |  | 0.836 |
| Yes | 96.2% (125/130) |  |
| No | 96.6% (198/205) |  |

Abbreviations: mITT, modified intention-to-treat; P-CAB, potassium-competitive acid blocker; PP, per-protocol.

**Table S2. The demographic information and clinical characteristics of patients** **who underwent and did not undergo gastroscopy**

| **Factors** | **With gastroscopy**  **(*n* = 292)** | **Without gastroscopy**  **(*n* = 76)** | ***P*** |
| --- | --- | --- | --- |
| Sex (male:female) | 106:186 | 36:143 | 0.254 |
| Age, y | 51 (44-58) | 50 (38-55.5) | 0.026 |
| BMI, kg/m^2^ | 23.1 (20.0-26.0) | 23.4 (21.2-25.2) | 0.651 |
| BSA, m^2^ | 1.64 (1.55-1.74) | 1.66 (1.59-1.78) | 0.093 |
| Smoking | 62 (21.2%) | 7 (9.2%) | 0.017 |
| Drinking (≥1 time per month) | 52 (17.8%) | 13 (17.1%) | 0.886 |
| Combined chronic diseases | 96 (32.9%) | 32 (42.1%) | 0.132 |
| Family history of gastric cancer | 12 (4.1%) | 1 (1.3%) | 0.409 |
| History of penicillin allergy | 18 (6.2%) | 7 (9.2%) | 0.347 |
| Digestive symptoms | 188 (64.4%) | 12 (15.8%) | ＜0.001 |
| Abdominal distension | 97 (33.2%) | 5 (6.6%) | ＜0.001 |
| Acid reflux | 73 (25.0%) | 1 (1.3%) | ＜0.001 |
| Belching | 65 (22.3%) | 2 (2.6%) | ＜0.001 |
| Abdominal pain | 63 (21.6%) | 0 | ＜0.001 |
| Upper abdominal discomfort | 54 (18.5%) | 1 (1.3%) | ＜0.001 |
| Heartburn | 42 (14.4%) | 2 (2.6%) | 0.009 |
| Nausea | 12 (4.1%) | 0 | 0.138 |
| Number of previous failed eradication treatments |  |  | 0.026 |
| 2 | 135 (46.2%) | 48 (63.2%) |  |
| 3-4 | 139 (47.6%) | 26 (34.2%) |  |
| ≥5 | 18 (6.2%) | 2 (2.6%) |  |

**Table S3. The demographic information and clinical characteristics of patients** **with and without tested antimicrobial resistance genes**

| **Factors** | **Tested**  **(*n* = 143)** | **Untested**  **(*n* = 225)** | ***P*** |
| --- | --- | --- | --- |
| Sex (male:female) | 50:93 | 89:136 | 0.376 |
| Age, y | 51 (43-58) | 50 (43-58) | 0.967 |
| BMI, kg/m^2^ | 23.8 (20.2-26.6) | 23.1 (20.2-25.1) | 0.209 |
| BSA, m^2^ | 1.66 (1.55-1.72) | 1.64 (1.56-1.77) | 0.412 |
| Smoking | 29 (20.3%) | 40 (17.8%) | 0.549 |
| Drinking (≥1 time per month) | 31 (21.7%) | 34 (15.1%) | 0.107 |
| Combined chronic diseases | 47 (32.9%) | 81 (36.0%) | 0.539 |
| Family history of gastric cancer | 12 (3.5%) | 1 (3.7%) | 1.000 |
| History of penicillin allergy | 8 (5.6%) | 17 (7.6%) | 0.466 |
| Digestive symptoms | 86 (60.1%) | 114 (50.7%) | 0.075 |
| Abdominal distension | 51 (35.7%) | 51 (22.7%) | 0.007 |
| Acid reflux | 44 (30.8%) | 30 (13.3%) | ＜0.001 |
| Belching | 34 (23.8%) | 33 (14.7%) | 0.027 |
| Abdominal pain | 28 (19.6%) | 35 (15.6%) | 0.318 |
| Upper abdominal discomfort | 26 (18.2%) | 29 (12.9%) | 0.165 |
| Heartburn | 27 (18.9%) | 17 (7.6%) | 0.001 |
| Nausea | 9 (6.3%) | 3 (1.3%) | 0.021 |
| Number of previous failed eradication treatments |  |  | 0.410 |
| 2 | 65 (45.5%) | 118 (52.4%) |  |
| 3-4 | 69 (48.3%) | 96 (42.7%) |  |
| ≥5 | 9 (6.3%) | 11 (4.9%) |  |
| Previously used bismuth quadruple therapy | 139 (97.2%) | 213 (94.7%) | 0.368 |
| Previously used triple therapy | 31 (21.7%) | 40 (17.8%) | 0.355 |
| Previously used high-dose dual therapy | 17 (11.9%) | 19 (8.4%) | 0.278 |
| Previously used clarithromycin | 137 (95.8%) | 208 (92.4%) | 0.194 |
| Previously used levofloxacin | 33 (23.1%) | 68 (30.2%) | 0.134 |

**Table S4. Adverse events of vonoprazan-based vs. tegoprazan-based regimen**

| **Safety** | **vonoprazan**  **(*n* = 326)** | **tegoprazan**  **(*n* = 42)** | ***P*** |
| --- | --- | --- | --- |
| Patients with adverse events | 84 (25.8%) | 11 (26.2%) | 0.953 |
| Dizziness | 31 (9.5%) | 4 (9.5%) | 1.000 |
| Nausea | 17 (5.2%) | 2 (4.8%) | 1.000 |
| Abdominal distension | 16 (4.9%) | 3 (7.1%) | 0.806 |
| Abdominal pain | 10 (3.1%) | 1 (2.4%) | 1.000 |
| Vomiting | 10 (3.1%) | 0 | 0.612 |
| Diarrhea | 8 (2.5%) | 2 (4.8%) | 0.718 |
| Fatigue | 8 (2.5%) | 0 | 0.604 |
| Rash | 5 (1.5%) | 0 | 1.000 |
| Headache | 5 (1.5%) | 0 | 1.000 |
| Insomnia | 5 (1.5%) | 0 | 1.000 |

**Table S5. Adverse events of different doses of colloidal bismuth pectin**

| **Safety** | **200 mg twice daily**  **(*n* = 301)** | **150 mg three times daily**  **(*n* = 67)** | ***P*** |
| --- | --- | --- | --- |
| Patients with adverse events | 83 (27.6%) | 12 (17.9%) | 0.102 |
| Dizziness | 31 (10.3%) | 4 (6.0%) | 0.389 |
| Nausea | 16 (5.3%) | 3 (4.5%) | 1.000 |
| Abdominal distension | 17 (5.6%) | 2 (3.0%) | 0.558 |
| Abdominal pain | 10 (3.3%) | 1 (1.5%) | 0.690 |
| Vomiting | 10 (3.3%) | 0 | 0.219 |
| Diarrhea | 6 (2.0%) | 4 (6.0%) | 0.163 |
| Fatigue | 7 (2.3%) | 1 (1.5%) | 1.000 |
| Rash | 4 (1.3%) | 1 (1.5%) | 1.000 |
| Headache | 4 (1.3%) | 1 (1.5%) | 1.000 |
| Insomnia | 5 (1.7%) | 0 | 0.590 |

**Table S6. Tolerance and Variance Inflation Factor (VIF) in the regression model**

| **Variables** | **Tolerance** | **VIF** |
| --- | --- | --- |
| Symptoms of nausea | 0.962 | 1.040 |
| Atrophic gastritis | 0.952 | 1.050 |
| Peptic ulcer | 0.990 | 1.010 |
| Previously used high-dose dual therapy | 0.549 | 1.821 |
| Previously used P-CABs-containing therapy | 0.540 | 1.853 |
| Adverse events | 0.892 | 1.122 |
| Poor compliance | 0.874 | 1.144 |

**Table S7. Collinearity diagnostics**

| **Dimension** | **Eigenvalue** | **Condition Index** | **Variance Proportion** | | | | | | | |
| --- | --- | --- | --- | --- | --- | --- | --- | --- | --- | --- |
|  |  |  | **Constant** | **Symptoms of nausea** | **Atrophic gastritis** | **Peptic ulcer** | **Previously used high-dose dual therapy** | **Previously used P-CABs-containing therapy** | **Adverse events** | **Poor compliance** |
| 1 | 4.837 | 1.000 | 0.00 | 0.00 | 0.01 | 0.01 | 0.00 | 0.01 | 0.00 | 0.00 |
| 2 | 1.372 | 1.878 | 0.00 | 0.00 | 0.00 | 0.00 | 0.16 | 0.15 | 0.00 | 0.00 |
| 3 | 0.881 | 2.343 | 0.00 | 0.00 | 0.00 | 0.99 | 0.00 | 0.00 | 0.00 | 0.00 |
| 4 | 0.478 | 3.181 | 0.00 | 0.00 | 0.95 | 0.00 | 0.02 | 0.00 | 0.01 | 0.00 |
| 5 | 0.302 | 4.002 | 0.00 | 0.00 | 0.01 | 0.00 | 0.77 | 0.80 | 0.00 | 0.00 |
| 6 | 0.074 | 8.076 | 0.02 | 0.05 | 0.00 | 0.00 | 0.00 | 0.01 | 0.95 | 0.03 |
| 7 | 0.043 | 10.626 | 0.03 | 0.15 | 0.02 | 0.00 | 0.02 | 0.04 | 0.03 | 0.90 |
| 8 | 0.013 | 19.076 | 0.94 | 0.79 | 0.00 | 0.00 | 0.00 | 0.00 | 0.01 | 0.07 |


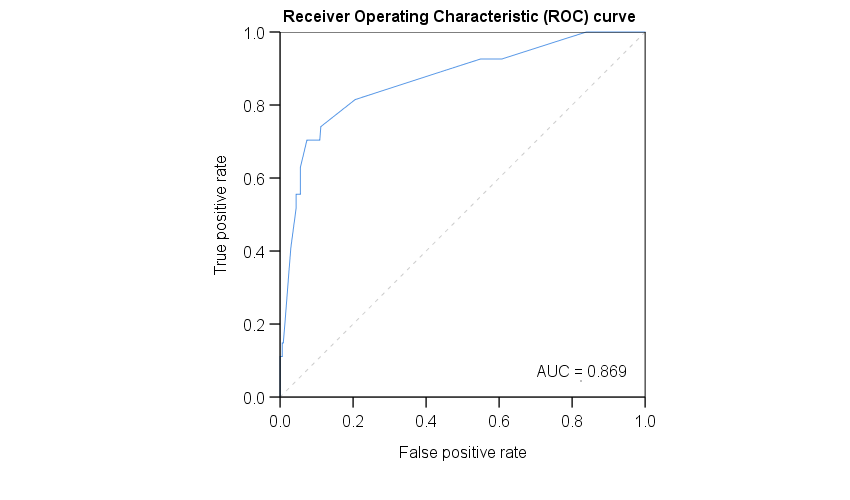


**Figure S1. The Receiver Operating Characteristic (ROC) curve of the logistic regression model**
